# Sexual activities and experiences in women who underwent genital cosmetic surgery: a cross-sectional study using data from the German Health and Sexuality Survey (GeSiD)

**DOI:** 10.1038/s41443-022-00621-0

**Published:** 2022-10-03

**Authors:** Thula U. Koops, Christian Wiessner, Peer Briken

**Affiliations:** 1 Institute for Sex Research, Sexual Medicine, and Forensic Psychiatry, University Medical Centre Hamburg-Eppendorf, Hamburg, Germany; 2 Institute of Medical Biometry and Epidemiology, University Medical Centre Hamburg-Eppendorf, Hamburg, Germany

**Keywords:** Health care, Medical research

## Abstract

The aim of this study was to compare women who have undergone genital cosmetic surgery (FGCS) with women who have not regarding past sexual activities and experiences. It draws on data from the German Health and Sexuality Survey (*GeSiD*). The subsample of women who had undergone FGCS (*n* = 32) was compared to a subsample of women who had not had FGCS (*n* = 96); the samples were matched for age, education, relationship and marital status, and whether participants had born a child. Variables concerning the present relationship, recent/lifetime sexual activities, sexual orientation, pregnancy-related experiences, health, sexual boundary violations/violence, sexual difficulties, and migration background served as main outcome measures. Women who had undergone FGCS reported more often anal intercourse during their last sexual encounter (13% vs. 1%, *p* = 0.021), a pregnancy ending in miscarriage (34% vs. 16%, *p* = 0.016), and not to be satisfied with their own appearance (41% vs. 15%, *p* = 0.002) than women who had not undergone FGCS. The results indicate women’s motivations for FGCS beyond the desire to improve genital appearance or function, and that contributing factors might be clinically relevant regarding more general psychological wellbeing.

## Introduction

The demand for genital cosmetic surgery in women (FGCS) such as the alteration of the labia (‘labiaplasty’) or vaginal ‘tightening’/‘rejuvenation’ has substantially increased over the last years, and is still on the rise: According to the Global Aesthetics Survey the worldwide increase of performed labiaplasties from 2016 to 2017 (1%) and of ‘vaginal rejuvenations’ (22%) taken together represented the largest of all reviewed procedures [1, 2]. In 2019 the number of reported labiaplasties worldwide (164,667) had grown by 24.1% compared to the year before, and by 73% compared to 2015 [3].

This trend has led experts from different fields to raise concerns about societal influences [4–7]—such as the medicalisation of sexuality, the regulative influence of public health practices, neoliberal imperatives around self-improvement, or negative sociocultural representations of female bodies/genitalia—and psychological vulnerabilities [8] amplifying the perceived urgency for and normalisation of surgery in women, especially with regard to online contents [9–11], as well as about the lack of data on surgery outcomes and side effects [12, 13], and regulations for providers [13]. Moreover, the (in some countries legally underpinned) distinction between practices known as ‘female genital cutting/mutilation’ (FGC/M) and FGCS, and the framing of the former as harmful and coercive and the latter as not have been ethically challenged [14–17].

Discontent with genital appearance in women and the consequential consideration of FGCS are well documented [18–20]. Motivations for undergoing FGCS can broadly be subsumed under ‘appearance’ (often described as aiming for ‘normality’ and self-confidence in sexual interactions) and ‘function’ (referencing sexual and nonsexual contexts) [18, 21, 22]. Likewise, improvement of sexual experience is an outcome frequently claimed by providers (e.g., [9, 23]). Not only has such a view on sexuality been criticised as “mechanical” [8], but a study by Krissi, Ben-Shitrit [24] showed no link between vulval anatomy and sexual ‘function’. A number of studies have reported increased levels of sexual ‘functioning’ and satisfaction after FGCS [22, 25–27]. However, in one study [22] only one item each assessed sexual ‘functioning’ and enjoyment, in others the level of sexual ‘functioning’ had fallen to baseline at the long-term follow-up [26, 27] and close to 50% of initial participants dropped out of the study after surgery [26]. Additionally, all studies were conducted in connection with the surgery, potentially affecting participants’ judgement of the outcome. To our knowledge, this is the first study to explore in more detail sexual activities and experiences in women who have undergone FGCS in a survey independent of the surgical procedure.

## Methods

Reporting of our study results is in line with STROBE guidelines [28].

### Dataset

This cross-sectional study is based on data from the German Health and Sexuality Survey (*GeSiD*). A doubly stratified residence registration office sample was collected in a two-step process, in which 200 sample points (step 1) and address data of 18- to 75-year-old residents from those sample points (step 2) were randomly selected. Participants were surveyed between October 2018 and September 2019 by interviewers from the social science research institute KantarEmnid with computer-assisted personal interviews (CAPI) involving a computer-assisted self-administered interview (CASI) part. In total, 4955 women and men participated in the survey, leading to a participation rate of 30.2% (AAPOR [American Association for Public Opinion Research] response rate 4, 29). All participants gave written informed consent. The survey instrument had been developed and tested in a preliminary study [30]; its final version consisted of 264 items. However, due to numerous filter variables, the number of items participants filled out varied based on their sexual and relationship experiences. Therefore, interview duration ranged between 19 and 208 min (51 min on average). To assess bias through non-response, demographic differences between responders and non-responders were ruled out by means of comparative analysis, or accounted for in the weighting procedure. For a detailed description of methods and outcomes see Matthiesen, Pietras [31].

For this analysis, we drew a subsample from the *GeSiD* dataset consisting of women who had answered “yes” to the item “Have you ever had aesthetic surgical procedures (cosmetic surgery) in the genital area performed on you?” (referred to as “FGCS”; *n* = 32). We then created a control sample from the *GeSiD* dataset of women who had answered “no” to this item (referred to as “no FGCS”; *n* = 96), matching this case sample regarding age, education, relationship and marital status, and whether participants had born a child (yes/no) (see Table 1 for sample characteristics).

**Table 1:** Sample characteristics.

		FGCS (*n* = 32)	no FGCS (*n* = 96)
Age [Mean (SD)]		45.97 (15.62)	46.13 (15.17)
Education [%]	No degree/9 years of school 10 years of school 12 or 13 years of school/university degree	15.6 37.5 46.9	15.6 37.5 46.9
Relationship status [%]	Not in a relationship In a relationship with a man	15.6 84.4	15.6 84.4
Marital status [%]	Single Married Widowed Divorced	25.0 53.1 3.1 18.8	25.0 53.1 3.1 18.8
Born a child [%]	Yes	71.9	71.9
Migration background [%]	Yes	22	21

Ethical approval for the *GeSiD* study was granted by the ethics committee of the Hamburg Psychotherapy Association (*Psychotherapeutenkammer Hamburg*; reference number 07/2018-PTK-HH). The study was funded by a grant from the German Federal Centre for Health Education (*BZgA*).

### Items and instruments

For the present study, we included items on the topics presented in Table 2.

**Table 2:** Survey topics and items.

Migration background		
Migration background	“What is your citizenship”	German/other
	“Which of the following persons was born abroad and immigrated to Germany or held foreign citizenship at birth?”	1: myself
Relationship		
Duration	“How long have you been together with (…)?”	Years; months
Age of partner	“How old is (…)”?	Years
Relationship satisfaction	“Altogether, how satisfied are you currently with your relationship?”	1: not satisfied at all – 7: completely satisfied
Duration of singlehood if not in relationship	“For how long have you now been without a steady partner?”	Years; months
Recent sexual activities		
Most recent sexual activities with partner	“When was the last time you had sex with (…)?”	1: today; 2: in the last week; 3: in the last 4 weeks; 4: in the last 3 months; 5: in the last 6 months; 6: in the last 12 months; 7: more than a year ago
Frequency in the past four weeks	“In total, how often did you have sex in the last 4 weeks with (…)?”	approximate number
Avoidance of sex	“Were there situations in the past 12 months in which you consciously avoided sex? If yes, how often did this happen?”	1: never; 2: rarely; 3: sometimes; 4: frequently; 5: very frequently
Sexual activities during last sex	“Please think of the last time you had sex with (…).What did you do that time?”	multiple answers possible: 1: vaginal intercourse; 2: oral intercourse; 3: anal intercourse; 4: other genital contacts
Orgasm during last sex	“Did you have an orgasm that last time?”	1: no; 2: yes, one time; 3: yes, several times; 8: don’t remember (anymore)
Satisfaction with sex life	“All things considered, I am satisfied with my sex life”	1: completely disagree – 5: Completely agree
Masturbation in the last 4 weeks/12 months	“How often did you masturbate in the last 12 months”?	1: never; 2: one time or several times in the last year; 3: one time or several times in the last 4 weeks; 4: several times per week; 5: daily
	“How often did you masturbate in the last 4 weeks?”	number
Lifetime sexual activities		
Sex outside the relationship	“Have you ever had sex with another person since you’ve been together with (…)?”	no; yes, with (number) person(s)
Sexual orientation	“Who do you feel sexually attracted to?”	1: exclusively men; 2: predominately men; 3: men and women; 4: predominately women; 5: exclusively women; 6: neither men nor women
	“Please choose the answer describing best how you think of yourself at the moment. I am …”	1: exclusively heterosexual; 2: predominately heterosexual; 3: bisexual; 4: predominately lesbian; 5: exclusively lesbian; 6: asexual; 7: other (specify)
Number of sexual partners	“In total, how many different men/women have you had sex with so far (meaning in your entire lifetime)?”	number
Pregnancy-related experiences		
Lifetime pregnancy	“Have you ever been pregnant?”	1: no; 2: yes
Lifetime miscarriage(s)	“Not every pregnancy ends with the birth of a child. What was it in your case: How did your respective pregnancies end?”	3: miscarriages: “How many miscarriages?” (number)
General health		
General health	“How is your general state of health?”	1: very good; 2: good; 3: average; 4: bad; 5: very bad
In treatment for depression	“In the last 12 months, have you been in treatment (therapy) for one of the following diseases?”	7: depression
Satisfaction with overall bodily appearance	“How satisfied are you currently with your appearance?”	1: very satisfied; 2: satisfied; 3: unsatisfied; 4: very unsatisfied
Sexual difficulties		
Difficulties regarding sexual desire, arousal, orgasm, and pain, pelvic floor muscle tension, or fear of pain	“Please indicate for each of the following experiences whether you have felt them over the course of several months in your life. (a) I had no or considerably reduced sexual desire or a considerably reduced drive to engage in sexual activity. (b) My response to sexual stimuli was absent or significantly reduced. (c) I only rarely had an orgasm, or my orgasm was less intense or delayed. "(d) had significant—persistent or recurrent—difficulties during sexual intercourse, for example because of tension of the pelvic floor muscles, pain, or fear of pain.”	1: no; 2: yes (These items stem from a screener for sexual ‘dysfunction’ as defined by the ICD-11 guidelines; see Briken, Matthiesen [51], Pietras, Wiessner [52] for instrument details)
Experiences of sexual boundary violations/violence		
Experiences of non-consensual touch	“Have you ever had another person attempting to sexually touch you against your will or to make you touch them?”	1: no; 2: yes, this happened or was attempted
Experiences of non-consensual intercourse	“Have you ever had another person have oral, anal, or vaginal sex (sexual intercourse) with you against your will or attempt to do so?”	1: no; 2: yes, this happened or was attempted

All items included the option to “not specify” to prevent non-response to items. Variables with multiple response categories in which some categories had been chosen by too few participants were transformed into dichotomous variables to allow for statistical analysis. All items included in Table 2 served as dependent variables in the analysis.

### Statistical analysis

We employed a matching of identified FGCS cases to controls with a ratio of 1:3, based on age, education, relationship and marital status. Differences in continuous variables were tested by a paired samples *t*-test from a mixed model with pair being the random effect. Differences in categorical variables were tested by stratified cross-tabulations with pair being the stratum variable. These methods were used to account for the dependency of the data due to the matching procedure. *P* values <0.05 were considered statistically significant; given the exploratory character and low power of the study due to the small sample size, we chose to report *p* values <0.20 as potentially relevant to avoid missing possible differences between persons who underwent FCGS and the control group. Moreover, for the same reasons we refrained from further examination of relationships between the dependent variables. The matching was conducted with the R package matchIt (version 3.6.2). Statistical tests were computed with SAS software version 9.4 (SAS Institute, Cary, NC).

## Results

Results of the comparison between women with and without experiences with FGCS are presented in Table 3. Women with experiences with FGCS indicated significantly more often to have engaged in anal intercourse during their last sexual encounter (13% in the FCGS groups vs. 1% in the No FCGS group), to have experienced a pregnancy ending in miscarriage (34% FCGS vs. 16% No FCGS), and not to be satisfied with their own appearance (41% FCGS vs. 15% No FCGS). Among the remaining variables, scores did not significantly differ between the groups, but a *p-*value <0.20 was obtained by the comparison of vaginal intercourse (87% FCGS vs. 97% No FCGS), oral intercourse (48% FCGS vs. 31% No FCGS), and other genital contacts (35% FCGS vs. 20% No FCGS) during the last sexual encounter, a treatment of depression in the last year (19% FCGS vs. 9% No FCGS), lifetime experiences of non-consensual intercourse (9% FCGS vs. 22% No FCGS), and of sexual pain (16% FCGS vs. 25% No FCGS).

**Table 3:** Group comparison (paired samples t-test and Mantel-Haenszel method).

	FGCS (*n* = 32)	no FGCS (*n* = 96)	*p* value
Relationship duration in years [Mean (SD)]	17.4 (13.8)	17.4 (15.8)	0.996^a^
Age of partner in years [Mean (SD)]	44.7 (15.9)	47.2 (14.3)	0.455^a^
Relationship satisfaction [Mean (SD)]	5.7 (1.3)	5.7 (1.2)	0.955^a^
Last sex during this week [%]	62	57	0.720
Frequency of sex in past 4 weeks [Mean (SD)]	4.0 (4.8)	3.8 (4.0)	0.809^a^
Avoidance of sex at least sometimes [%]	46	38	0.535
Vaginal intercourse during last sex [%]	87	97	0.071
Oral intercourse during last sex [%]	48	31	0.101
Anal intercourse during last sex [%]	13	1	**0.021**
Other genital contacts during last sex [%]	35	20	0.070
Orgasm during last sex [%]	70	70	0.636
Satisfaction with sex life [Mean (SD)]	4.0 (1.2)	3.8 (1.2)	0.481
Sex outside current relationship [%]	23	21	1.000
More than 5 years single [%]	6	10	0.206
Completely or mainly attracted to men [%]	97	91	0.273
Completely or mainly heterosexual [%]	97	91	0.236
Number of sexual partners [Mean (SD)]	9.1 (10.9)	8.1 (10.5)	0.647^a^
Masturbation in past 12 months [%]	71	64	0.407
Masturbation in past 4 weeks [Mean (SD)]	2.1 (3.3)	2.7 (6.2)	0.623^a^
Pregnancy lifetime [%]	75	75	0.706
Abortion lifetime [%]	16	11	0.411
Miscarriage lifetime [%]	34	16	**0.016**
General health good/very good [%]	72	71	0.906
Treatment of depression in last 12 months, %	19	9	0.160
Not satisfied with own appearance [%]	41	15	**0.002**
Sexual arousal problems lifetime [%]	35	47	0.288
Orgasmic problems lifetime [%]	41	51	0.317
Sexual pain lifetime [%]	16	25	0.161
Non-consensual sexual touch lifetime [%]	38	42	0.673
Non-consensual intercourse lifetime [%]	9	22	0.096

bold: *p *< 0.05.

*SD* standard deviation.

^a^Paired samples *t*-test.

## Discussion

The comparison revealed differences with respect to several variables which might offer further insights into influential factors in the pursuit of FGCS. The extent to which the more frequent engagement in anal intercourse during the last sexual encounter indicates a difference in preference for or enjoyment of anal sexual stimulation needs to be explored in further detail, especially considering the predominating neglect of pleasure from heterosexual anal sex in existing research [32]. However, available studies found that men rated the experience of anal intercourse as significantly more pleasurable than women [33], that women had engaged in anal intercourse following their partner’s request [34], pressure or coercion [35, 36], and that anal intercourse in women was associated with having participated in sexual intercourse unwillingly [37, 38] and with supporting views around male dominance [38]. In correspondence with the description of FGCS as a way of creating ‘prototypical’ genitalia to counter perceived ‘abnormality’ or gender ambiguity [39, 40], a more frequent engagement in anal intercourse could also point to a tendency in women with FGCS to adhere to societal norms on gender and sexual agency (e.g., men being the initiators of sexual activities and women the ‘gatekeepers’; or women having to cater to men’s sexual ‘needs’ or ‘entitlement’ to sex). The interconnectedness of gender-related norms and individual (biographic) aspects which has shown to be relevant for example for the experience of sexual pain in women [41] and its effect on the desire for FGCS needs to be investigated by future studies. Beyond that, it needs to be explored if a more frequent engagement in anal intercourse expresses a particular attitude towards the women’s own vulva, since descriptively women with FGCS also had engaged less often in vaginal and more often in oral sex and other genital contacts; an avoidance of sexual practices leading to pregnancy; or, as mentioned above, a difference in pleasure from anal intercourse.

An involvement of sexual trauma in women’s desire to surgically alter their genitalia is not supported by our data, as women with FGCS had not experienced sexual boundary violations or sexual violence more often than women from the control sample. Still, sexual harassment and objectification of women’s bodies can occur in more subtle forms than hands-on behaviour, which is why more detailed—preferably qualitative—investigations on this matter need to be conducted.

In line with our results, Ålgars, Santtila [42] found a connection between pregnancies not carried to term and sexual body image: women who reported miscarriages expressed more dissatisfaction with their breasts than women who did not; women who had had an abortion were more dissatisfied with both their breasts and their genital appearance. Following the authors’ conclusion, it is possible that such experiences lead to a more negative perception of one’s own body, particularly with regard to the parts associated with reproduction; or that a more negative body image increases the likelihood of such experiences. In light of the potential influence of gender-norm adherence mentioned above, an interesting question for future research is if and how this aspect could be involved in negative (and positive) perceptions of one’s own ‘reproductive’ body. To add another parallel, the experience of pregnancy loss has shown links with depression [43–45], which as well has been associated with body image dissatisfaction [46]—the third characteristic in which groups in this analysis differed—and the pursuit of cosmetic surgery [47, 48]. A treatment for depression in the last year had indeed been reported by a share of women twice as large in the “FGCS” as in the “no FGCS” group (a nearly statistically significant difference). The fact that women with experiences with FGCS in this study more often reported not being satisfied with their appearance in general gives rise to the question of whether a broader difficulty with accepting and relating to one’s body is involved in the dissatisfaction with genital appearance. It is conceivable that there is an interaction between past depressive complaints and general body image dissatisfaction, and experiences of pregnancy loss, drawing attention to reproduction-associated body parts and culminating in a desire for FGCS.

In addition to this, the similarities between the groups regarding the majority of variables, including most sexual behaviours and lifetime experience of sexual difficulties—with arousal, orgasm, and pain problems even being descriptively less prevalent among women who had undergone FGCS—, could support the view that the desire for FGCS is less likely to arise from a particular sexual ‘lifestyle’ or problem which requires FGCS. This is of course speculative given the limited information in our data on how participants experience sexual activities. Nevertheless, it casts doubt on the common presentation of sexual ‘functioning’ and satisfaction are presented as central motivators for FGCS. A combined qualitative investigation into women’s perception and understanding of their sexual behaviour, and their motivations for FGCS is required to develop further hypotheses on links between them. Thereby, existing evidence for the impact of media exposure (for example to advertisement or sexually explicit media) and of negative feedback from intimate partners or peers on women’s motivations for FGCS [49, 50] could be extended, considering that these factors might influence sexual behaviour and experience as well.

### Strengths and limitations

A limitation of the results of this study is the fact that the experience of FGCS was only queried using one dichotomous item. For a more in-depth interpretation of our data, it would have been useful to have more information on the type of surgery that was performed, what motivations for undergoing FGCS participants name, how satisfied they had been with the result or whether they had experienced any complications post-surgery. Given that the *GeSiD* survey encompassed items on a great variety of topics and had to be kept at a reasonable length for participants, going into more detail was not feasible. Furthermore, as the *GeSiD* survey represented a cross-sectional study, and no time frame was requested for many experiences—including FGCS—, our data did not allow for a causal interpretation of the links detected in the analysis. Considering the large number of statistical tests we conducted and the consequential risk of a type 1 error, the differences found need to be validated by future studies. Nonetheless, a major strength of the dataset is that the survey included items on a wide range of sexuality- and body-related topics and did not prime participants to answer with regard to the impact of their cosmetic surgery. As participants were not recruited via their provider of FGCS, a bias through a focus on the effects of FGCS on sexuality was avoided. Moreover, the data stem from a subsample of a representative sample of German women, for which reason the results can be considered more robust than those from analyses of clinical samples.

## Conclusion

The present study provides insights into sexual activities and experiences of women with experiences with FGCS, which give directions for future investigations. Several factors were identified as potentially relevant for the pursuit of FGCS; the role of these factors needs to be studied in more detail, ideally with both qualitative and prospective approaches. Although the generalisability of the results is restricted by the small sample size and lacking information on timing and types of procedures, the results indicate that women’s motivations for FGCS might go beyond the desire to improve genital appearance or function, and that contributing factors might also be relevant for the more general psychological wellbeing of women who opt for FGCS procedures.

## Data Availability

The data that support the findings of this study are available from the corresponding author, [TUK], upon reasonable request.
